# Anatomical description of malformations of the neck of the left atrial appendage

**DOI:** 10.1002/ca.24246

**Published:** 2024-11-14

**Authors:** Jakub Batko, Rafał Jakiel, Agata Krawczyk–Ożóg, Kacper Jaśkiewicz, Radosław Litwinowicz, Marian Burysz, Marcin Jakiel, Krzysztof Bartuś, Filip Bolechała, Marcin Strona, Mateusz Krystian Hołda

**Affiliations:** ^1^ HEART—Heart Embryology and Anatomy Research Team, Department of Anatomy Jagiellonian University Medical College Cracow Poland; ^2^ CAROL—Cardiothoracic Anatomy Research Operative Lab, Department of Cardiovascular Surgery and Transplantology, Institute of Cardiology Jagiellonian University Medical College Cracow Poland; ^3^ Thoracic Research Centre, Collegium Medicum Nicolaus Copernicus University, Innovative Medical Forum Bydgoszcz Poland; ^4^ Department of Cardiology and Cardiovascular Interventions University Hospital in Cracow Cracow Poland; ^5^ Department of Cardiac Surgery Regional Specialist Hospital Grudziądz Poland; ^6^ Department of Cardiovascular Surgery and Transplantology, Institute of Cardiology Jagiellonian University Medical College Cracow Poland; ^7^ Department of Forensic Medicine Jagiellonian University Medical College Cracow Poland; ^8^ Division of Cardiovascular Sciences The University of Manchester Manchester UK

**Keywords:** atrial appendage, atrial fibrillation, clinical anatomy, computed tomography, left atrial appendage, left atrial appendage closure, left atrium

## Abstract

The recently‐described left atrial appendage (LAA) neck is a truncated cone‐shaped structure that connects the LAA orifice to its lobe. It shows malformations in some cases, but their exact description and clinical significance are unknown. Therefore, the aim of this study was to provide a detailed anatomical and morphometric analysis of LAA neck malformations in clinical context. A total of 250 autopsied human hearts (20.0% women, 46.7 ± 18.2 years old) were examined for mural malformations: spikes and bulges. Endocardial roughness of the LAA neck with a depth <2 mm and no recognizable epicardial protrusion was defined as ectopic trabeculation. LAA neck malformations were found in 13.6%, bulges in 10.0% of the hearts examined, spikes in only 3.2%, and ectopic trabeculations in 24.8%. In one case, both a bulge and a spike were found in the LAA neck. Most LAA neck roughness was observed on the aortic and venous surfaces of the LAA neck. Those surfaces were the most common locations for malformations and ectopic trabeculations. The LAA wall was significantly thinner than the surrounding neck wall within the bulges and the ectopic trabeculations, but not in the spikes.

## 1 INTRODUCTION

In view of the frequency of left atrial appendage (LAA) exclusion, recent years have seen a growing interest in the anatomy of the LAA (Batko et al., 2023; Naksuk et al., 2016; Slodowska et al., 2022; Słodowska et al., 2021; Whiteman et al., 2019). LAA exclusion can be performed in patients with atrial fibrillation for whom oral anticoagulation is contraindicated, with the aim of eliminating the main cardiac source of thrombi in the human body (Hindricks et al., 2021; Steffel et al., 2021; Whitlock et al., 2021). Exclusion can be performed in three ways: surgically sutured (closed), surgically removed, or percutaneously occluded. Various devices have been developed to simplify these procedures and enhance their safety (Burysz et al., 2019; Grygier et al., 2018; Lee & Hanke, 2022; Litwinowicz et al., 2021; Toale et al., 2019). Percutaneous LAA occlusion can be achieved through endoluminal device implantation (e.g., using the Watchman FLX (Boston Scientific) or Amulet (Abbott) devices) or by percutaneous ligation with a suture using the LARIAT (SentreHEART) device (Grygier et al., 2018; Hindricks et al., 2021). Potential complications associated with these procedures include pericardial effusion, cardiac perforation, procedure‐related stroke, device embolization, and device‐related thrombus formation (Burysz et al., 2019; Litwinowicz et al., 2021; Reddy et al., 2013; Whitlock et al., 2021).

The LARIAT method is distinctive, combining percutaneous access for implanting the ligature suture stem with percutaneous epicardial placement of the LARIAT device in order to place a stitch around the base of the LAA (Batko et al., 2023; Litwinowicz et al., 2023). Its main advantage over other endocardial methods is that no foreign body is implanted into the LAA. In principle, this eliminates the risk of device‐related thrombus and device embolism, which, although rare, are serious complications of endocardial LAA closure. Additionally, access complications associated with venous puncture and transseptal puncture are specific to percutaneous LAA closures and are absent in the LARIAT procedure. However, the procedure can still be challenging, particularly in patients with specific anatomical variants of the LAA (Batko et al., 2022; Grygier et al., 2018; Wang et al., 2010).

For epicardial LAA closure during cardiac surgery an AtriClip device can be used, which requires only epicardial access. However, this is exclusively performed as part of another surgical procedure (Lee & Hanke, 2022; Litwinowicz et al., 2019; Toale et al., 2019). Its main weakness is that the clip placed on the LAA can slip and injure vital structures in the vicinity, including the aorta, pulmonary trunk, coronary arteries, pulmonary veins, walls of the left atrium, or mitral valve annulus. It can also lead to pericarditis in rare cases (Toale et al., 2019). Nevertheless, it provides a simple and feasible option for LAA closure in various types of anatomy that are not always suitable for other LAA exclusion procedures.

One determining factor in choosing an LAA exclusion technique is the anatomy of the LAA (Batko et al., 2022; Slodowska et al., 2022; Słodowska et al., 2021; Wang et al., 2010). The LAA can be divided anatomically into orifice, neck and lobe (Batko et al., 2023). This subdivision is essential for understanding the clinical implications of the LAA and the region‐specific challenges involved in selecting an appropriate LAA exclusion procedure. The lobe is the central part of the LAA body, characterized by pectinate muscles. The recently‐described LAA neck is a truncated cone‐shaped structure that connects the LAA orifice to the lobe (Batko et al., 2023). The walls of the LAA neck are mostly smooth and homogeneous, whereas the walls of the LAA lobe are significantly thinner and lined with pectinate muscles (Batko et al., 2023; Slodowska et al., 2022). Although the anatomy of the LAA body is relatively well known, the LAA neck has not yet been fully described (Batko et al., 2023; Naksuk et al., 2016; Slodowska et al., 2022; Słodowska et al., 2021). There are malformations of the smooth neck walls in some cases, but their exact description and clinical significance are unknown; there are no descriptions of such malformations in the literature.

Therefore, the aim of this study was to provide a detailed anatomical and morphometric analysis of LAA neck malformations. We also attempted to place our observations in clinical context, particularly in relation to epicardial and endocardial LAA exclusion procedures.

## 2 MATERIALS AND METHODS

This study was approved by the Bioethical Committee of the Jagiellonian University, Kracow, Poland (No. 1072.6120.205.2019 and 1072.6120.4.2020). The study protocol conforms to the ethical guidelines of the 1975 Declaration of Helsinki. The authors state that every effort was made to follow all local and international ethical guidelines and laws that pertain to the use of human cadaveric donors in anatomical research (Iwanaga et al., 2022).

### 2.1 Study population

A total of 250 autopsied human hearts were analyzed (46.7 ± 18.2 years old, mean BMI 26.3 ± 4.1 kg/m^2^ and mean BSA 1.9 ± 0.2 m^2^, 80.0% of males and 20.0% females). The samples were collected during standard forensic autopsies at the Department of Forensic Medicine, Jagiellonian University Medical College, Krakow, Poland. The most common causes of death were suicide, homicide and traffic accidents. The exclusion criteria were as follows: History of cardiac surgery, conspicuous severe macroscopic pathologies of the heart or vascular system detected at autopsy, cardiac trauma, and macroscopic signs of decomposition of the corpse. Hearts were routinely removed from the chest cavity and fixed by immersion in a 10% paraformaldehyde buffer solution pending observations and measurements.

### 2.2 Definitions and measurements

**FIGURE 1:**
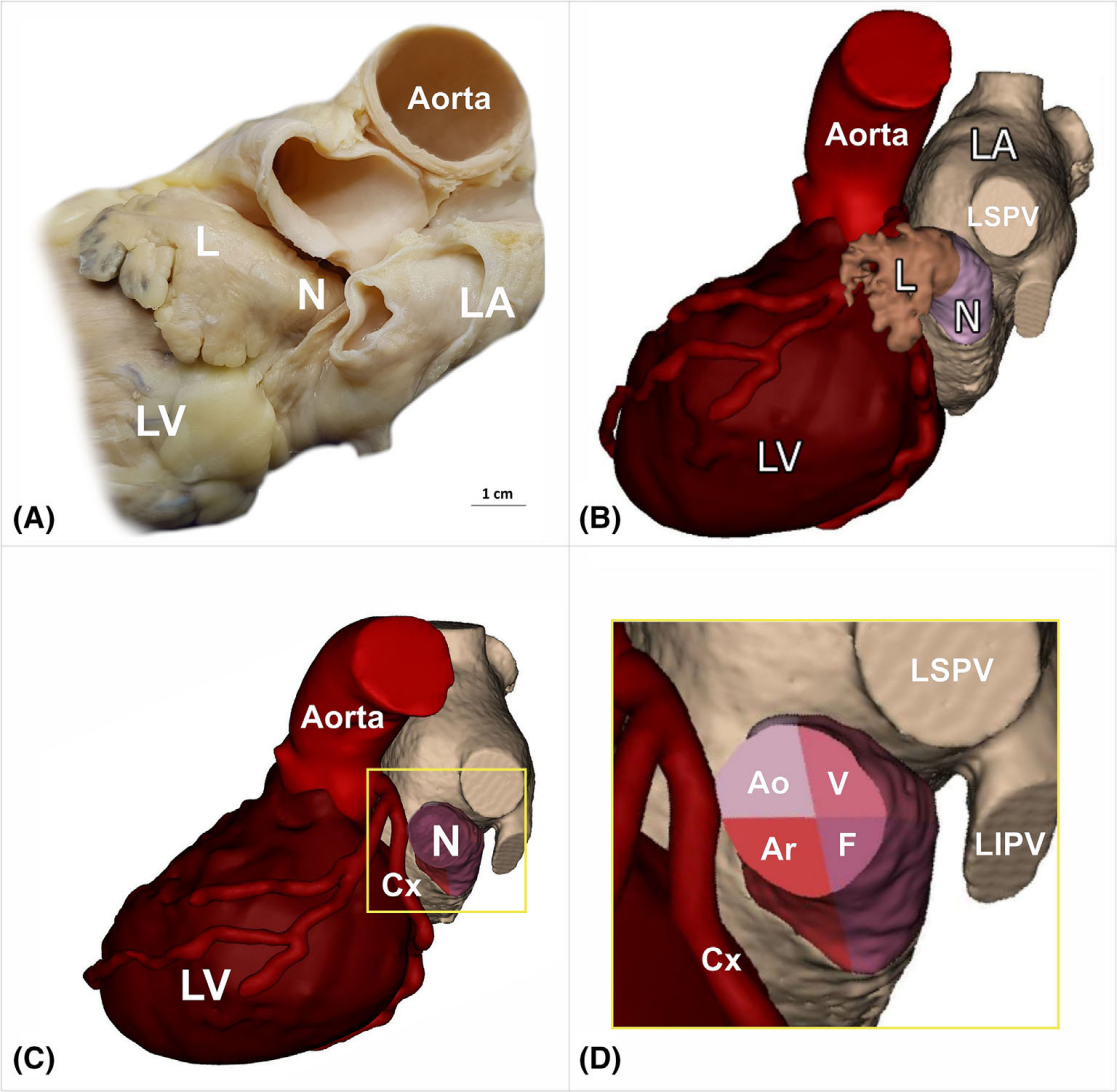
Location of the left atrial appendage (LAA) neck and its wall subdivision—a left lateral view of the left atrial area. (A) Photograph of cadaveric heart specimens showing the LAA region with the visible LAA neck (N) and lobe (L). (B) Three‐dimensional visualization of the heart, highlighting the LAA region and showing a clear division of the LAA into the lobe and neck. (C) Three‐dimensional visualization of the LAA neck and its surrounding structures, with the LAA body removed to reveal the division of the LAA neck into four distinct surfaces. (D) Magnified view of the selected area in the adjacent panel. The LAA neck is subdivided into four surfaces: Venous (V), aortic (Ao), arterial (Ar), and free (F). Three‐dimensional reconstructions of the heart subcomponents were generated from contrast‐enhanced, electrocardiogram‐gated computed tomography scans using volume‐rendering and segmentation techniques in Innovation Suite 23 (Materialize, Belgium). Cx, left circumflex artery; LA, left atrium; LIPV, left inferior pulmonary vein; LSPV, left superior pulmonary vein; LV, left ventricle.

The shape of the LAA lobe was determined using a previously‐established simplified classification system with three types (cauliflower, chicken wing, and arrowhead) (Słodowska et al., 2021). To inspect the LAA neck, the left atrium was opened in a standard fashion through an incision between the pulmonary vein ostia. The LAA neck was defined as a frustoconical canal bounded proximally by the LAA orifice and distally by the lobe entrance (Grygier et al., 2018). As previously described, the LAA neck can be further subdivided into four surfaces:

- venous—adjacent to the ostium of the left superior pulmonary vein,
- aortic—adjacent to the aortic root,
- arterial—adjacent to the left circumflex artery,
- and the free surface lying between the venous and arterial surfaces (Figure 1) (Batko et al., 2023).

**FIGURE 2:**
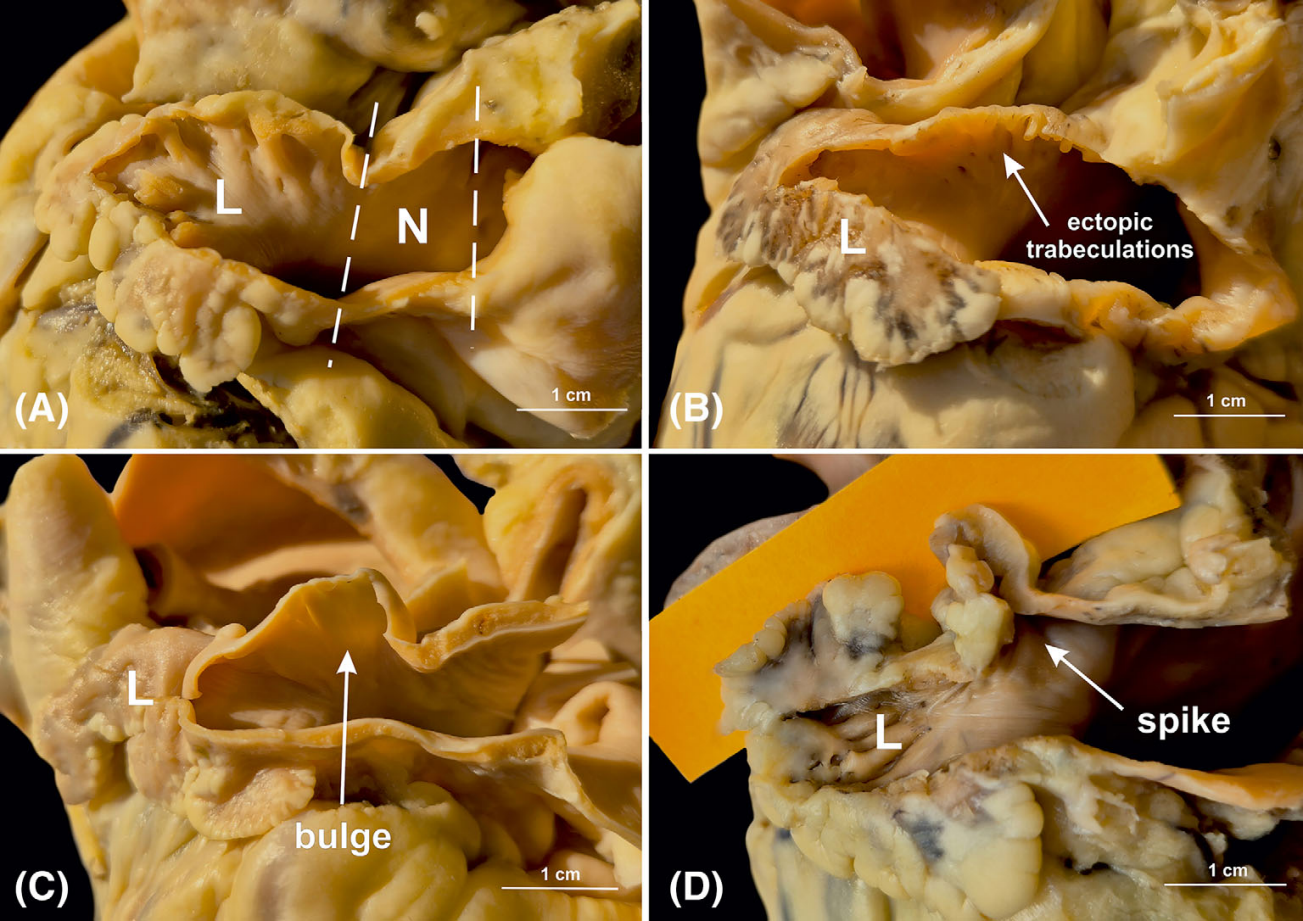
Photographs of cadaveric heart specimens displaying the neck region of the left atrial appendage (LAA). One of the LAA walls was removed to provide a clearer view of the interior. (A) LAA with no malformations (smooth walls). (B) Cross‐section showing ectopic trabeculations (surface roughness without a discernible epicardial protrusion). (C) Cross‐section showing a bulge (semi‐curved epicardial protrusions with a shallow lumen and a large endocardial opening). (D) Cross‐section showing a spike (elongated epicardial projections with a deep lumen and a small endocardial opening). L, lobe of the LAA; N, neck of the LAA.

**FIGURE 3:**
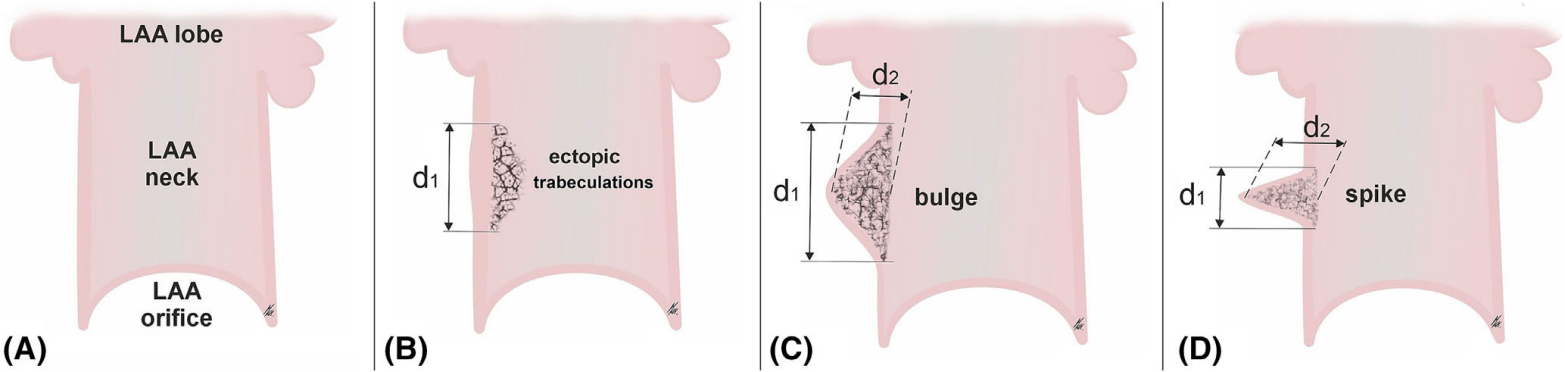
Schematic diagrams of different types of the left atrial appendage (LAA) neck malformations. (A) LAA without malformations (smooth endocardial wall), (B) ectopic trabeculation (surface roughness without a recognizable epicardial protrusion), (C) bulge (semi‐curved epicardial protrusion with a shallow lumen and large endocardial opening), (D) spike (elongated epicardial projection with deep lumen and small endocardial opening). The measurement sites are indicated (d1–diameter, d2–depth; see details in section 2).

Each surface of the LAA neck was examined for wall malformations and ectopic trabeculations on the endocardial surface (Figure 2). Malformations were defined as endocardially‐visible roughness of the LAA neck wall—pectinate trabeculations forming islands of depressions (at least 2 mm deep) consisting of mesh‐like clefts and diverticula against the smooth surface of the surrounding endocardium (Figure 3). From the epicardial aspect, the surface roughness was expressed as a significant protrusion on the surface of the cone. Two types were distinguished:

- bulges—semi‐curved epicardial protrusions with a shallow lumen and large endocardial opening (Figure 3C);
- spikes—elongated epicardial projections with deep lumen and small endocardial opening (Figure 3D).

If the LAA neck showed endocardial roughness but with a depth <2 mm and no recognizable epicardial protrusion, it was defined as an ectopic trabeculation (Figure 3B).

For better visualization and measurement, the LAA neck was cut longitudinally between the venous and free or venous and aortic surfaces. The exact location of each LAA neck malformation was noted and the longitudinal and transverse dimensions of the endocardial roughness were measured. The depth of each malformation was also measured. The surface of each LAA neck was cut open in the center to obtain a transverse section that allowed us to measure the wall thickness (endocardium + myocardium). Thickness was also measured at the bottom of the malformation and at 10 randomly selected points within the LAA lobe.

Two independent researchers obtained all the measurements using electronic calipers with a metrological threshold of 0.03 mm (YT‐7201, YATO, Poland). In each case, the mean of the two measured values was reported. The area of the malformations was calculated using the formula for the area of an ellipse. If the measurements differed by more than 10% between the researchers, they were repeated.

### 2.3 Statistical analysis

Data were analyzed using IBM SPSS Statistics 29.0 (Predictive Solutions). Categorical variables are presented as numbers (*n*) or percentages. Quantitative variables are presented as mean with standard deviation. Normality of distribution was assessed using the Shapiro–Wilk test. Differences between normally‐distributed parameters were assessed using the Student *t*‐test, and non‐normally distributed quantitative data were tested using the Mann–Whitney U‐test. Differences between categorical variables were assessed using the chi‐square test for independence. Differences between more than two groups were also assessed. For normally distributed data, one‐way analysis of variance (one‐way ANOVA) with Tukey's post‐hoc test was used if the ANOVA results were statistically significant. For non‐normally distributed data, the Kruskal and Wallis test with Dunn's post‐hoc test was used if the Kruskal and Wallis test gave statistically significant results. Correlations were tested using the rho‐Spearman correlation (two‐tailed, α = 0.05, β = 0.2). *p* values for the post‐hoc tests of the direct group comparison are presented in the results. The *p*‐values for the ANOVA for the simultaneous group comparison are shown in the tables. A *p*‐value <0.05 was considered statistically significant.

## 3 RESULTS

There were LAA neck malformations in 13.6% of the hearts examined, bulges in 10.0% and spikes in only 3.2%. In one case (0.4%), both a bulge and a spike were found. There were reticular chords and tissue bridges in every malformation examined. Ectopic trabeculations without malformations of the LAA neck wall were observed in 24.8% of hearts (lesions less than 2 mm deep), co‐occurring with bulges in four cases (1.6%) and with two separate ectopic trabeculations in one (0.4%). Table 1 details the prevalence and locations of LAA neck malformations and ectopic trabeculations. The aortic and venous surfaces were the most common locations for both malformations and ectopic trabeculations. There were no significant differences in the distribution of malformations and ectopic trabeculations among the different LAA types (cauliflower, chicken wing, and arrowhead), and no associations with anthropometric parameters (sex, age, body weight, height, body mass index).

**TABLE 1:** The localization of left atrial appendage (LAA) malformations and ectopic trabeculations.

Localization at the LAA neck surface	LAA malformations *n* = 34*		Ectopic trabeculations *n* = 62	*p*‐value cumulative	*p*‐value bulge vs. spike	*p*‐value bulge vs. ectopic trab.	*p*‐value spike vs. ectopic trab.
	Bulge *n* = 25	Spike *n* = 8					
Aortic	2 (8.0%)	3 (37.5%)	30 (48.4%)	0.019	0.043	<0.001	0.562
Venous	7 (28.0%)	3 (37.5%)	12 (19.4%)	0.415	0.611	0.377	0.239
Free	0 (0.0%)	0 (0.0%)	4 (6.5%)	‐	‐	‐	‐
Arterial	0 (0.0%)	0 (0.0%)	0 (0.0%)	‐	‐	‐	‐
Venous/aortic border	13 (52.0%)	2 (25.0%)	10 (16.1%)	0.003	0.181	<0.001	0.531
Arterial/free border	3 (12.0%)	0 (0.0%)	6 (9.7%)	‐	‐	0.748	‐

* In one case both a bulge and a spike were found in the LAA neck (excluded from further analyses).

Table 2 shows the morphometric characteristics of the neck malformations and ectopic trabeculations. There were several significant differences in the sizes of the lesions (bulges vs. spikes vs. ectopic trabeculations) (Table 2). The ectopic trabeculations had the largest area, followed by bulges and spikes (77.6 ± 42.6 vs. 69.2 ± 50.4 vs. 26.9 ± 11.5 mm^2^, *p* < 0.001). The spikes were significantly deeper than the bulges (7.5 ± 0.7 vs. 4.7 ± 1.0 mm, *p* < 0.001). LAA neck lesion dimensions did not differ significantly between sexes or between LAA types (cauliflower, chicken wing, and arrowhead). Body weight correlated significantly with the transverse (r = 0.354, *p* = 0.042) and longitudinal (r = 0.485, *p* = 0.002) diameters of the ectopic trabeculations, and age correlated significantly with the transverse diameter (r = 0.437, *p* = 0.003).

**TABLE 2:** Morphometry of malformations and ectopic trabeculations of the left atrial appendage (LAA) neck (mean ± SD).

	General	LAA types			*p*‐value ANOVA for LAA types
		Cauliflower	Chicken wing	Arrowhead	
Bulge longitudinal diameter (mm)	10.7 ± 3.3	12.5 ± 5.7	11.0 ± 2.8	9.7 ± 3.4	0.418
Bulge transverse diameter (mm)	7.6 ± 3.1	10.9 ± 7.2	7.7 ± 2.4	6.3 ± 1.0	0.426
Bulge surface area (mm^2^)	69.2 ± 50.4	123.0 ± 119.2	68.4 ± 35.4	48.5 ± 22.6	0.415
Bulge depth (mm)	4.7 ± 1.0	4.7 ± 1.6	4.7 ± 1.1	4.7 ± 0.9	0.953
Spike longitudinal diameter (mm)	7.2 ± 1.3	7.4 ± 0.9	7.8 ± 1.7	6.2 ± 1.1	0.861
Spike transverse diameter (mm)	4.6 ± 1.1	5.2 ± 1.6	4.2 ± 1.2	4.0 ± 1.0	0.632
Spike surface area (mm^2^)	26.9 ± 11.5	31.2 ± 17.5	25.7 ± 16.4	19.5 ± 9.7	0.582
Spike depth (mm)	7.5 ± 0.7	7.7 ± 0.9	7.4 ± 0.3	7.1 ± 0.6	0.861
Ectopic trabeculation longitudinal diameter (mm)	9.7 ± 2.4	11.7 ± 4.6	10.3 ± 3.1	8.2 ± 2.7	0.517
Ectopic trabeculation transverse diameter (mm)	8.6 ± 4.1	9.2 ± 5.2	8.8 ± 4.4	5.9 ± 2.5	0.329
Ectopic trabeculation surface area (mm^2^)	77.6 ± 42.6	89.5 ± 49.2	76.2 ± 47.1	44.0 ± 19.3	0.211

Detailed data on LAA wall thicknesses at all measured sites are presented in Table 3. The wall is clearly thinner at the LAA lobe than at the LAA neck (*p* < 0.001). Within the LAA neck, the arterial surface has the thickest wall, followed by the aortic, free, and venous surfaces (Table 3, *p* < 0.001). The LAA wall within the bulges (0.5 ± 0.2 mm) and the ectopic trabeculations (0.8 ± 0.3 mm), but not in the spikes (1.1 ± 0.2 mm), is significantly thinner than the surrounding LAA neck wall. There were also significant differences in ectopic trabeculations wall thickness among LAA types (cauliflower < chicken wing < arrowhead, Table 3, *p* < 0.05). LAA wall thickness did not correlate significantly with anthropometric parameters (sex, age, body weight, height, BMI) at any measured site.

**TABLE 3:** Thickness of the left atrial appendage (LAA) walls at different sites (mean ± SD).

	General	LAA type			*p*‐value ANOVA for LAA types
		Cauliflower	Chicken wing	Arrowhead	
LAA lobe wall thickness (mm)	0.7 ± 0.2	0.7 ± 0.3	0.7 ± 0.2	0.8 ± 0.3	0.802
LAA lobe wall thickness with trabeculations (mm)	1.1 ± 0.2	1.3 ± 0.3	1.0 ± 0.3	1.1 ± 0.2	0.743
LAA neck venous surface wall thickness (mm)	1.0 ± 0.3	1.0 ± 0.3	0.9 ± 0.3	1.1 ± 0.4	0.975
LAA neck aortic surface wall thickness (mm)	1.2 ± 0.3	1.1 ± 0.3	1.3 ± 0.3	1.2 ± 0.4	0.965
LAA neck arterial surface wall thickness (mm)	1.9 ± 0.5	2.0 ± 0.6	1.9 ± 0.5	1.8 ± 0.5	0.704
LAA neck free surface wall thickness (mm)	1.1 ± 0.3	1.0 ± 0.3	1.1 ± 0.2	1.2 ± 0.2	0.361
Bulge wall thickness (mm)	0.5 ± 0.2	0.5 ± 0.1	0.5 ± 0.2	0.6 ± 0.3	0.723
Bulge wall thickness with trabeculations (mm)	0.8 ± 0.2	0.8 ± 0.2	0.7 ± 0.2	0.9 ± 0.3	0.454
Spike wall thickness (mm)	1.1 ± 0.2	1.2 ± 0.1	0.8 ± 0.3	1.0 ± 0.3	0.223
Spike wall thickness with trabeculations (mm)	1.3 ± 0.3	1.5 ± 0.1	1.2 ± 0.3	1.2 ± 0.2	0.264
Ectopic trabeculation wall thickness (mm)	0.8 ± 0.3	0.5 ± 0.2	0.7 ± 0.3	1.2 ± 0.4	0.019*
Ectopic trabeculation wall thickness with trabeculations (mm)	1.1 ± 0.2	0.7 ± 0.2	0.9 ± 0.3	1.4 ± 0.5	0.034*

* *p* value significant difference between cauliflower and arrowhead LAA types.

## 4 DISCUSSION

When discussing the LAA, its structure, function and clinical significance, we should be aware of its morphological division into two separate parts: lobes and neck (Batko et al., 2023; Slodowska et al., 2022; Słodowska et al., 2021). In recent years, the LAA lobe has dominated this structural analysis, leading to the development of several anatomical classifications of the LAA, including the most recent simplified classification (Słodowska et al., 2021). The main basis for this approach was the suspicion that certain morphological shapes of the LAA favor blood flow congestion, especially in patients with atrial fibrillation; this was supported by biomechanical flow analyzes in reconstructed LAAs (Naksuk et al., 2016). In addition, prospective investigations, including HEART‐CLOT studies, provided deep insights into the systemic effects of the LAA on hypercoagulability (Bartus et al., 2020; Litwinowicz et al., 2022). The LAA neck has received considerably less attention, as it has primarily been defined in clinical practice as a landing zone for LAA closure and occlusion devices (Batko et al., 2022; Grygier et al., 2018; Slodowska et al., 2022). It is the most proximal part of the LAA, separated from the left atrium by the LAA orifice, and is currently the focus of intensive research owing to its significance for optimal device placement and the potential for lacerations, which can lead to severe negative treatment outcomes (Batko et al., 2022; Fauchier et al., 2018; Grygier et al., 2018; Litwinowicz et al., 2023; Mhanna et al., 2021; Steffel et al., 2021). In the current study, we found that LAA neck malformations (13.6%) and ectopic trabeculations (24.8%) are relatively common lesions, collectively detected on the endocardial surface of the LAA neck in almost 40% of cases. These LAA endocardial lesions are solitary, focal, and limited in size. Their clinical significance remains undetermined.

One critical factor for successful endocardial LAA occlusion is the proper fitting of the occluder to the walls of the LAA neck (Grygier et al., 2018). Failure to achieve this can result in peri‐device leakage, leading to device‐related thrombus or even device embolism over a prolonged period. The regular, usually cylindrical, shape of the LAA occluder is designed for the standard LAA neck geometry (Batko et al., 2023). Malformations of the LAA neck, particularly wide bulges, can create enough space for leakage to occur, even after a correctly‐performed closure. Owing to the morphometry of the bulges, which resemble the LAA lobe more than the neck (with significantly thinner walls and trabeculations at the apex), adjusting the occluder to eliminate leakage can increase the risk of rupture in this vulnerable area. Conversely, spikes within the LAA neck can complicate the implantation of endoluminal devices, though their morphometry does not alter the overall endocardial geometry of the LAA neck significantly. During epicardial LAA closure, proper propagation of the LARIAT device or clip to the landing zone is critical for the success of the procedure (Litwinowicz et al., 2019; Litwinowicz et al., 2021). In this context, wide but shallow protrusions are less problematic than narrow, highly elevated spikes, which can obstruct the passage of instruments or risk catching and tearing during closure.

Current research raises the question of how to differentiate between small accessory LAAs in the LAA neck region and LAA neck malformations. In the literature, accessory appendages are defined as protrusions with a distinct narrow ostium, visible neck, and body, with irregular contours resembling pectinate muscles (Hołda et al., 2017; Ratusznik et al., 2024; Yoshihara et al., 2021). Similar structures can be found in the main cavity of the left atrium; they are known as left atrial diverticula, sac‐like structures with a broad ostium and a smooth body contour (Yoshihara et al., 2021). Morphologically, the bulge‐type LAA neck malformation closely resembles a left atrial diverticulum, but it is distinguished by the pectinate muscles. A spike‐type malformation can be misinterpreted as an accessory LAA; however, its regular shape, which is atypical for accessory LAAs, allows for clear differentiation.

Knowledge of the thickness of each LAA neck wall is crucial for understanding which regions are more susceptible to rupture or fistula formation due to the radial forces exerted by occluders or clips, and the potential penetrating forces from hooks. The arterial surface, adjacent to the left circumflex coronary artery, is the thickest part of the LAA neck. The thinnest surfaces are located on the opposite side, near the aortic root and the ostia of the pulmonary veins. This variation in wall thickness can increase the risk of rupture on the venous and aortic surfaces shortly after the procedure. Meanwhile, the thicker arterial surface could be subjected to greater force from closure devices over time, leading to fistula formation, which is most commonly observed in this region following endocardial LAA closure procedures (Batko et al., 2022; Katona et al., 2015; Mhanna et al., 2021).

**FIGURE 4:**
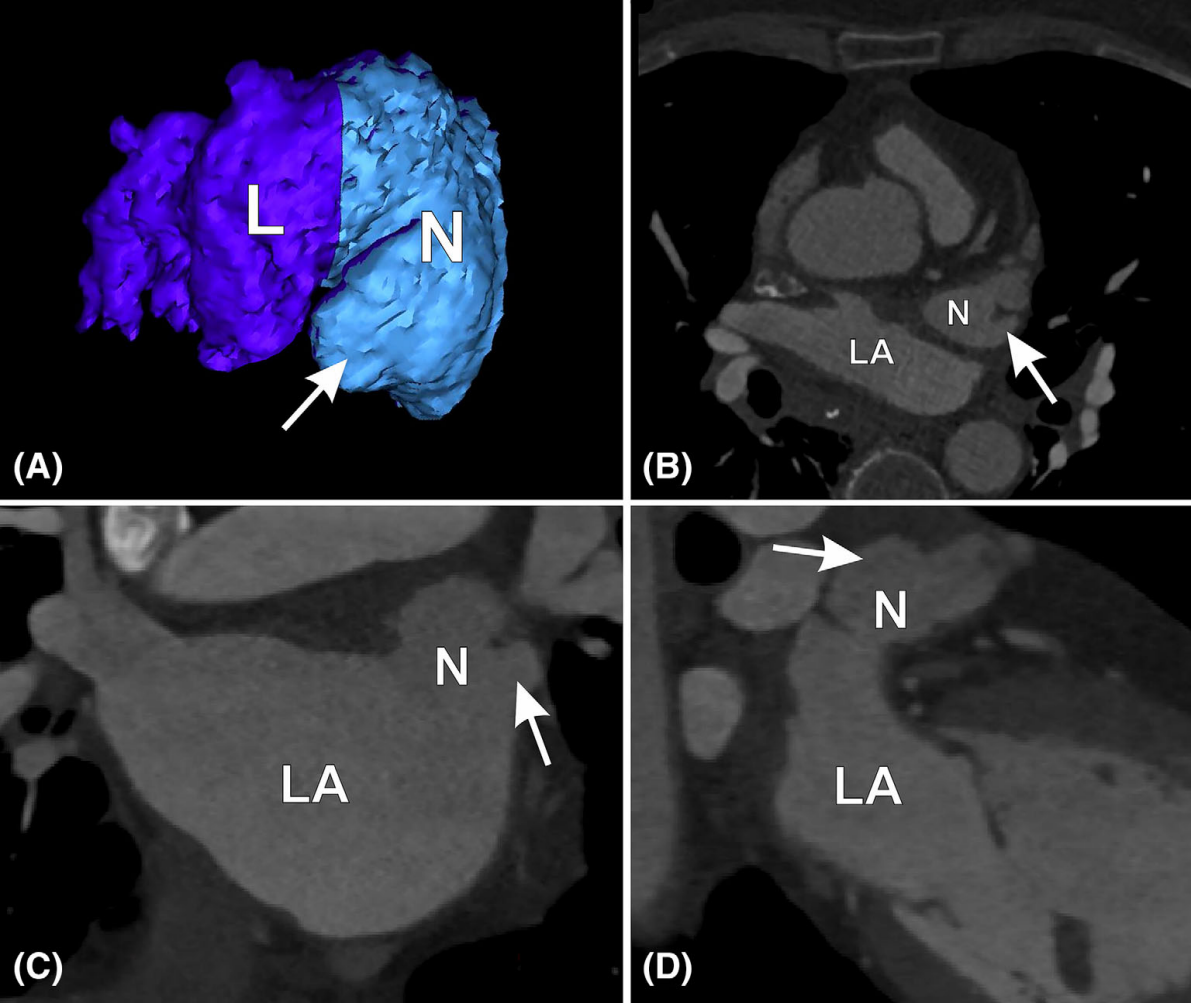
Cardiac computed tomography three‐dimensional reconstruction and multiplane visualizations of the same left atrial appendage (LAA) neck malformation (white arrows–bulge type). Pictures are generated from contrast‐enhanced, electrocardiogram‐gated computed tomography scans using volume‐rendering and segmentation techniques in Innovation Suite 23 (Materialize, Belgium). (A) Three‐dimensional reconstruction of the LAA. (B) Axial view. (C) Coronal view. (D) Sagittal view. L, lobe of the LAA; LA, left atrium; N, neck of the LAA.

LAA malformations can readily be visualized preoperatively using computed tomography angiography (Figure 4) (Abbara et al., 2009; Batko et al., 2023; Hołda et al., 2017; Slodowska et al., 2022; Słodowska et al., 2021). Transesophageal echocardiography can also be used to visualize LAA neck lacerations, but this requires optimal examination conditions and an experienced cardiac sonographer for detailed imaging of the LAA neck region (Bansal & Kasliwal, 2012). In future, standardized methods for detecting and measuring LAA malformations in imaging techniques should be developed. Given the morphological descriptions of LAA malformations in the current study, we suggest the following recommendations for implementation in daily clinical practice:

- During preoperative visualization of the LAA neck, the presence of LAA neck malformations should be routinely assessed.
- A bulge‐like malformation could make epicardial LAA closure preferable. Given their relatively high frequency, a cutoff size for bulge type LAA neck malformations should be established for this recommendation.
- A spike‐type malformation could make endocardial LAA occlusion the preferred option.
- If an LAA neck malformation is detected during LAA occlusion, extreme caution is advised in view of wall thinning in the malformation region, and rapid or forced movements in the LAA neck area should be avoided.

Several limitations of this study should be considered. First, because this is an autopsy study, we were not able to evaluate physiological changes in the LAA components during the cardiac cycle. The organs were fixed in paraformaldehyde, which could have influenced our morphometric observations. However, our previous studies have shown that the use of formaldehyde for tissue preservation did not change the cardiac dimensions significantly (Hołda et al., 2018). In addition, this study was conducted on a population in which females were underrepresented (20%) and only one ethnicity was studied (Caucasian). Finally, no representative individuals with atrial fibrillation (which could significantly affect LAA remodeling) were studied. Despite these limitations, we believe that our study provides valuable insights into the complex morphology of the LAA.

## 5 CONCLUSIONS

LAA neck malformations and ectopic trabeculations are relatively common lesions on the endocardial surface of the LAA neck, occurring in 13.6% and 24.8% of hearts, respectively. LAA endocardial lesions are solitary and focal, with limited dimensions. Most LAA neck roughness is found on the aortic and venous surfaces of the LAA neck. The LAA wall is significantly thinner within bulges and ectopic trabeculations than the surrounding LAA neck wall. Further studies should investigate the clinical implications of these anatomical features, as they could be associated with specific intraoperative and postoperative complications during LAA procedures.

## FUNDING INFORMATION

This research was supported by National Centre for Research and Development, Poland (LIDER/7/0027/L‐10/18/NCBR/2019). Funding sources had no involvement in study design, collection, analysis and interpretation of data, in writing of the report and in the decision to submit the article for publication.

## Data Availability

Data is available from corresponding author upon reasonable request.
